# Influenza evolution and H3N2 vaccine effectiveness, with application to the 2014/2015 season

**DOI:** 10.1093/protein/gzw017

**Published:** 2016-07-20

**Authors:** Xi Li, Michael W. Deem

**Affiliations:** 1 Department of Bioengineering, Rice University, Houston, TX 77005, USA; 2 Department of Physics and Astronomy, Rice University, Houston, TX 77005, USA; 3 Center for Theoretical Biological Physics, Rice University, Houston, TX 77005, USA

**Keywords:** dominant strains, influenza evolution, *p*_epitope_, phylogeny, vaccine effectiveness

## Abstract

Influenza A is a serious disease that causes significant morbidity and mortality, and vaccines against the seasonal influenza disease are of variable effectiveness. In this article, we discuss the use of the *p*_epitope_ method to predict the dominant influenza strain and the expected vaccine effectiveness in the coming flu season. We illustrate how the effectiveness of the 2014/2015 A/Texas/50/2012 [clade 3C.1] vaccine against the A/California/02/2014 [clade 3C.3a] strain that emerged in the population can be estimated via *p*_epitope_. In addition, we show by a multidimensional scaling analysis of data collected through 2014, the emergence of a new A/New Mexico/11/2014-like cluster [clade 3C.2a] that is immunologically distinct from the A/California/02/2014-like strains.

## Introduction

Influenza is a highly contagious virus, usually spread by droplet or fomite transmission. The high mutation and reassortment rates of this virus lead to significant viral diversity in the population (Ferguson *et al*., 2003; Rambaut *et al*., 2008). In most seasons and regions, one type of influenza predominates among infected people, typically A/H1N1, A/H3N2 or B. In the 2014/2015 season, A/H3N2 was the most common (Flannery *et al*., 2015). While there are many strains of influenza A/H3N2, typically there is a dominant cluster of strains that infect most people during one winter season. Global travel by infected individuals leads this cluster of sequences to dominate in most affected countries in a single influenza season. New clusters arise every 3–5 years by the combined effects of mutation and selection (Smith *et al*., 2004; He and Deem, 2010). There is significant selection pressure upon the virus to evolve due to prior vaccination or exposure (Illingworth *et al*., 2014; Łuksza and Lässig, 2014).

Due to evolution of the influenza virus, the strains selected by the World Health Organization (WHO) for inclusion in the seasonal vaccine are reviewed annually and often updated. The selection is based on which strains are circulating, the geographic spread of circulating strains and the expected effectiveness of the current vaccine strains against newly identified strains (http://www.cdc.gov/flu/professionals/vaccination/virusqa.htm, accessed 2 July 2015). There are to date 143 national influenza centers located in 113 countries that provide and study influenza surveillance data. Five WHO Collaborating Centers for Reference and Research on Influenza (Centers for Disease Control and Prevention, Atlanta, GA, USA; National Institute for Medical Research in London, UK; Victorian Infectious Diseases Reference Laboratory, Melbourne, Australia; National Institute of Infectious Diseases, Tokyo, Japan; and Chinese Center for Disease Control and Prevention, Beijing, China) are sent samples for additional analysis. These surveillance data are used to make forecasts about which strains are mostly likely to dominate in the human population. These forecasts are used by the WHO to make specific recommendations about the strains to include in the annual vaccine, in 2016 one each of a A/H1N1, A/H3N2 and influenza B Yamagata lineage or Victoria lineage subtype strain. Additionally, for each recommended strain there is often a list of 5–6 ‘like’ strains that may be substituted by manufacturers for the recommended strain and which may grow more readily in the vaccine manufacturing process that uses hen's eggs.

We here focus on predicting the expected effectiveness of the current vaccine strains against newly identified strains and on predicting or detecting the emergence of new influenza strains. Predicting effectiveness or emergence without recourse to animal models or human data is challenging. The influenza vaccine protects against strains similar to the vaccine but not against strains sufficiently dissimilar. For example, the A/Texas/50/2012(H3N2) 2014/2015 Northern hemisphere vaccine has been observed to not protect against the A/California/02/2014(H3N2) virus. Furthermore, there is no vaccine that provides long-lasting, universal protection, although this is an active research topic (Kanekiyo *et al*., 2013).

**Fig. 1:**
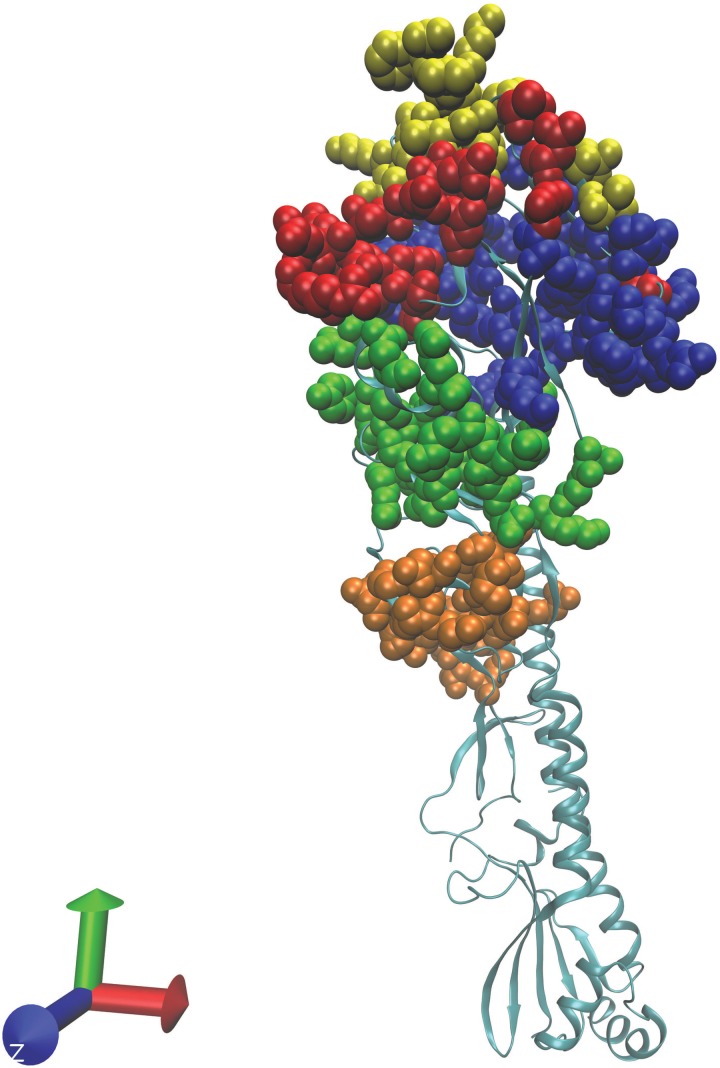
Shown is the structure of hemagglutinin in H3N2 (accession number 4O5N). The five epitope regions (Gupta *et al*., 2006) are color coded: epitope A is red (19 amino acids), B is yellow (21 aa), C is orange (27 aa), D is blue (41 aa), and E is green (22 aa). Note epitope B was dominant in 2013/2014 and 2014/2015.

Vaccine effectiveness is expected to be a function of ‘antigenic distance’. While antigenic distance is often estimated from ferret animal model hemagglutination inhibition (HI) studies, the concept is more general. In particular, in this study we are interested in the antigenic distance that the human immune system detects. A measurement of antigenic distance that is predictive of vaccine effectiveness for H3N2 and H1N1 influenza A in humans is *p*_epitope_ (see www.mwdeem.rice.edu/pepitope for a spreadsheet to calculate *p*_epitope_ values; Gupta *et al*., 2006; Pan and Deem, 2009, 2011; Pan *et al*., 2011). We show here that this approach correlates with H3N2 vaccine effectiveness in humans with *R*^2^ = 0.75. The quantity *p*_epitope_ is the fraction of amino acids in the dominant epitope region of hemagglutinin that differs between the vaccine and virus (Gupta *et al*., 2006). The structure of the H3N2 hemagglutinin is shown in Fig. 1, and the five epitopes are highlighted in color. The quantity *p*_epitope_ is an accurate estimate of influenza antigenic distance in humans. Previous work has shown that *p*_epitope_ correlates with influenza H3N2 vaccine effectiveness in humans with *R*^2^ = 0.81 for the years 1971–2004 (Gupta *et al*., 2006). While our focus here is H3N2, other work has shown that *p*_epitope_ also correlates with influenza H1N1 vaccine effectiveness in humans (Pan *et al*., 2011; Huang *et al*., 2012). The *p*_epitope_ measure has been extended to the highly pathogenic avian influenza H5N1 viruses (Peng *et al*., 2014). The *p*_epitope_ measure has additionally been extended to veterinary applications, for example equine H3N8 vaccines (Daly and Elton, 2013).

To determine the strains to be included in the vaccine, the emergence of new strains likely to dominate in the human population must be detected. We here use the method of multidimensional scaling to detect emerging strains. As an example, we apply the approach to the 2014–15 season. Dominant, circulating strains of influenza H3N2 in the human population typically have been present at low frequencies for 2–3 years before fixing in the population. While the frequencies of such emerging strains are low, they are high enough that samples are collected, sequenced and deposited in GenBank. Multidimensional scaling, also known as principal component analysis (Gower, 1967), has been used to identify clusters of influenza from animal model data (Smith *et al*., 2004). Thus, this method can be used to detect an incipient dominant strain for an upcoming flu season from sequence data alone, before the strain becomes dominant (He and Deem, 2010). We here use this method to detect emerging strains in the Northern Hemisphere 2014–15 season. Interestingly, H3N2 evolves such that the reconstructed phylogenetic tree has a distinct one-dimensional backbone (He and Deem, 2010; Strelkowa and Lässig, 2012).

In this article, we show that the current A/Texas/50/2012 vaccine is predicted not to protect against the A/California/02/2014 strain that has emerged in the population, consistent with recent observations (World Health Organization, 2014a). This A/California/02/2014 strain can be detected and predicted as a transition from the A/Texas/50/2012 strain. The proposed Southern Hemisphere summer 2015 and Northern Hemisphere 2015/2016 vaccine strain is A/Switzerland/9715293/2013, which is identical in the expressed hemagglutinin (HA1) region to the A/California/02/2014 strain. (There is one substitution each in epitopes A, B and D for the A/Switzerland/9715293/2013 E4/E2 strains.) Furthermore, we find that there is, in 2015/2016, a transition underway from the A/California/02/2014 cluster to an A/New Mexico/11/2014 cluster. The latter may be an appropriate vaccine component for Northern Hemisphere 2015/2016 season, because the new A/New Mexico/11/14 cluster is emerging and appears based upon representation in the sequence database to be displacing the A/California/02/14 cluster.

## Methods

### The *p*_epitope_ method

We calculate *p*_epitope_, the fraction of amino acids in the dominant epitope region of hemagglutinin that differ between the vaccine and virus (Gupta *et al*., 2006). We use epitope sites as in Gupta *et al*. (2006) and illustrate in Fig. 1. For each of the five epitopes (see www.mwdeem.rice.edu/pepitope for a spreadsheet to calculate *p*_epitope_ values; Gupta *et al*., 2006), we calculate the number of amino acid substitutions between the vaccine and virus and divide this quantity by the number of amino acids in the epitope. The value of *p*_epitope_ is defined to be the largest of these five values.

### Identification of vaccine strains and circulating strains

The dominant circulating influenza H3N2 strain and the vaccine strain were determined from annual WHO reports (World Health Organization, 2004, 2005, 2006a, b, 2007, 2008, 2009, 2010, 2011, 2012, 2013, 2014a, b). These strains are listed in Table I. In many years, the WHO report lists a preferred vaccine strain, while the actual vaccine is a ‘like’ strain. Additionally, in some years, different vaccines were used in different regions. For each study listed in Table I, the vaccine strain used is listed.

**Table I.** Historical vaccine strains, circulating strains and vaccine effectiveness

Year	Vaccine	Circulating strain	Dominant strain epitope	*P*_epitope_	Vaccine effectiveness	*n*_u_	*N*_u_	*n*_v_	*N*_v_	*d*_1_	*d*_2_
2004–05	A/Wyoming/3/2003 (AY531033)	A/Fujian/411/2002 (AFG72823)	B	0.095	9% (Ndifon *et al*., 2009)	6	40	50	367	2 (Huang, 2008)	1 (Huang, 2008)
2005–06	A/New York/55/2004 (AFM71868)	A/Wisconsin/67/2005 (AFH00648)	A	0.053	36% (Skowronski *et al*., 2007)	43	165	6	36	1 (World Health Organization, 2006a)	2 (World Health Organization, 2006a)
2006–07	A/Wisconsin/67/2005 (ACF54576)	A/Hiroshima/52/2005 (ABX79354)	A	0.105	5% (Skowronski *et al*., 2009)	130	406	20	66	1 (Bulimo *et al*., 2008)	2 (Bulimo *et al*., 2008)
2007	A/Wisconsin/67/2005 (ACF54576)	A/Wisconsin/67/2005 (AFH00648)	B	0.048	54% (Fielding *et al*., 2011)	74	234	8	55		
2008–09	A/Brisbane/10/2007 (ACI26318)	A/Brisbane/10/2007 (AIU46080)		0	51% (Simpson *et al*., 2015)	36	240	4	54		
2010–11	A/Perth/16/2009 (AHX37629)	A/Victoria/208/2009 (AIU46085)	A	0.053	39% (World Health Organization, 2011; Treanor *et al*., 2012)	100	991	35	569	0 (European Center for Disease Prevention and Control, 2011)	1.4 (European Center for Disease Prevention and Control, 2011)
2011–12	A/Perth/16/2009 (AHX37629)	A/Victoria/361/2011 (AIU46088)	C	0.111	23% (World Health Organization, 2012; Castilla *et al*., 2013)	335	616	47	112	1 (Martin *et al*., 2015)	2.8 (Martin *et al*., 2015)
2012–13	A/Victoria/361/2011 (AGB08328)	A/Victoria/361/2011 (AIU46088)	B	0.095	35% (Kissling *et al*., 2014)	288	1257	15	100	5 (World Health Organization, 2013)	4 (World Health Organization, 2013)
2013–14	A/Victoria/361/2011 (AGL07159)	A/Texas/50/2012 (AIE52525)	B	0.190	12% (Castilla *et al*., 2014)	145	476	16	60	5 (World Health Organization, 2013)	4 (World Health Organization, 2013)
2014–15	A/Texas/50/2012 (AIE52525)	A/California/02/2014 (AIE09741)	B	0.191	14% (Flannery *et al*., 2015)	135	342	100	293	4 (World Health Organization, 2014b)	5.6 (World Health Organization, 2014b)

H3N2 influenza vaccine effectiveness in humans and corresponding *p*_epitope_ antigenic distances for the 2004–15 seasons. The vaccine and circulating strains are shown for each of the years since 2004 in which H3N2 virus has been the predominant influenza virus and for which vaccine effectiveness data are available. Vaccine effectiveness values are taken from the literature. Here *N*_u_ is the total number of unvaccinated subjects, *N*_v_ is the total number of vaccinated subjects, *n*_u_ is the number of H3N2 influenza cases among the unvaccinated subjects and *n*_v_ is the number of H3N2 influenza cases among the vaccinated subjects. Also shown are the distances derived from ferret HI data by the two common measures (Gupta *et al*., 2006).

### Estimation of vaccine effectiveness

Vaccine effectiveness can be quantified. It is defined as (Gupta *et al*., 2006)

1 $$E=\frac{u-v}{u},$$

where *u* is the rate at which unvaccinated people are infected with influenza and *v* is the rate at which vaccinated people are infected with influenza.

The vaccine effectiveness in Equation (1) was calculated from rates of infection observed in epidemiological studies. Influenza H3N2 vaccine effectiveness values for years 1971–2004 are from studies previously collected (Gupta *et al*., 2006). Laboratory-confirmed data for the years 2004–15 were collected from the studies cited in Table I. Epidemiological data from healthy adults, aged ∼18–65, were used. For each study, the total number of unvaccinated subjects (*N*_u_), the total number of vaccinated subjects (*N*_v_), the number of H3N2 influenza cases among the unvaccinated subjects (*n*_u_) and the number of H3N2 influenza cases among the vaccinated subjects (*n*_v_) are known and listed in the table. From these numbers, vaccine effectiveness was calculated from Equation (1), where *u* = *n*_u_/*N*_u_ and *v* = *n*_v_/*N*_v_. Error bars, *ϵ*, on the calculated effectiveness values were obtained assuming binomial statistics for each data set (Gupta *et al*., 2006): *ϵ*^2^ = [*σ*^2^/*u*^2^/*N*v+ (*v*/*u*^2^)^2^*σ*^2^/*N*u], where $\sigma_{v}^{2}=v(1-v)$ and $\sigma_{u}^{2}=u(1-u)$.

### Virus sequence data in 2013 and 2014

The evolution of the HA1 region of the H3N2 virus in the 2013/2014 and 2014/2015 seasons was analyzed in detail. We downloaded from GenBank the 1006 human HA1 H3N2 sequences that were collected in 2013 and the 179 human HA1 H3N2 sequences that were collected in 2014.

### Sequence data alignment

All sequences were aligned before further processing by multialignment using Clustal Omega. Only full-length HA1 sequences of 327 amino acids were used, as partial sequences were excluded in the GenBank search criterion. Default clustering parameters in Clustal Omega were used. There were no gaps or deletions detected by Clustal Omega in the 2013 and 2014 sequence data.

### Multidimensional scaling

Multidimensional scaling finds a reduced set of dimensions that best reproduce the distances between all pairs of a set of points. In the present application, the points are HA1 sequences of length 327 amino acids, and the data were reduced to *n*= 2 dimensions. Distances between two sequences were defined as the Hamming distance, i.e. the number of differing amino acids, divided by the total length of 327. In this way, multidimensional scaling places the virus sequences in a reduced sequence space so that distances between pairs of viral sequences are maintained as accurately as possible. This low-dimensional clustering method enables one to visualize the viruses, by finding the two best dimensions to approximate the Hamming distances between all clustered sequences.

### Gaussian kernel density estimation

The method of Gaussian kernel density estimation was used to predict the probability density of sequences in the reduced sequence space identified by multidimensional scaling (He and Deem, 2010). Briefly, each sequence was represented by a Gaussian distribution centered at the position, where the sequence lies in the reduced space. The total estimated viral probability density was the sum of all of these Gaussians for each virus sequence. The weight of the Gaussian for each sequence was constant. The standard deviation of the Gaussian for each sequence was roughly one-half amino acid substitution in the dominant epitope, *σ* = 0.5/327. In other words, the reconstructed probability density of the viruses in the reduced (*x*, * y*) space, as estimated by the sequences from GenBank, was given by *p*(*x*, *y*) ∝ Σ*_i_*exp{−[(*x* − *x_i_*)^2^ + (*y* − *y_i_*)^2^]/2*σ*^2^)}, where the location of virus *i* in the reduced space is (*x_i_*, *y_i_*) and *σ* is the standard deviation. In this way, a smooth estimation of the underlying distribution of virus sequences from which the sequences deposited in GenBank are collected is generated.

There are three criteria by which a new cluster can be judged to determine if it will dominate in the human population in a future season. First, the cluster must be evident in a density estimation. Second, the cluster must be growing. That is, there must be evident selection pressure on the cluster. Third, the cluster must be sufficiently far from the current vaccine strain, as judged by *p*_epitope_, for the vaccine to provide little or no protection against the new strains. From prior work (Gupta *et al*., 2006) and from the results discussed below, peaks separated by more than roughly *p*_epitope_ = 0.19 are sufficiently separated that protection against the virus at one peak is expected to provide little protection against the viruses at the other.

## Results and discussion

### Vaccine effectiveness correlates with antigenic distance

**Fig. 2:**
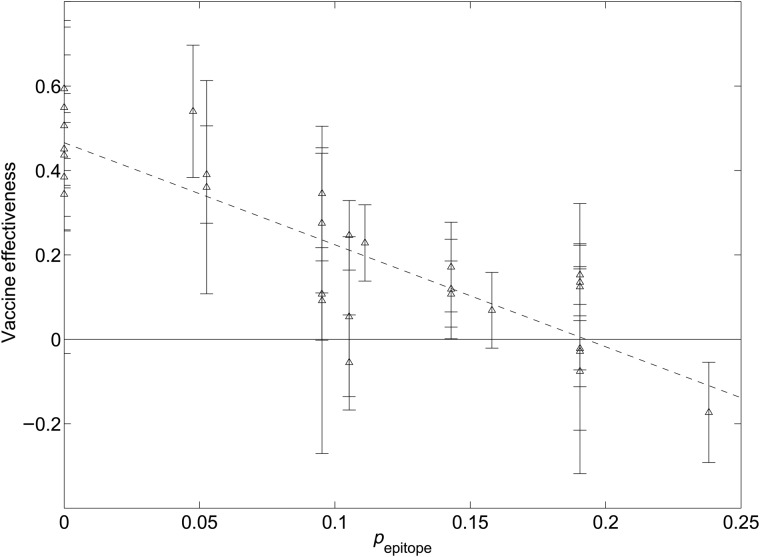
Vaccine effectiveness in humans as a function of the *p*_epitope_ antigenic distance. Vaccine effectiveness values from epidemiological studies of healthy adults, aged approximately 18–65, are shown (triangles). Also shown is a linear fit to the data (solid, *R*_2_ = 0.75). Vaccine effectiveness declines to zero at *p*_epitope_ = 0.19 on average. The error bars show the standard estimate of the mean of each sample point, as discussed in the text.

Figure 2 shows how vaccine effectiveness decreases with antigenic distance. The equation for the average effectiveness (the solid line in Fig. 2) is

$$E=-2.417\;p_{\mathrm{epitope}}+0.466.$$

Vaccine effectiveness declines to zero at approximately *p*_epitope_ > 0*.*19, on average. When the dominant epitope is A or B, in which there are 19 or 21 amino acids, respectively, this means that vaccine effectiveness declines to zero after roughly four substitutions. When the dominant epitope is C, in which there are 27 amino acids, the vaccine effectiveness declines to zero after roughly five substitutions.

Figure 2 shows that H3N2 vaccine effectiveness in humans correlates well with the *p*_epitope_ measure of antigenic distance. In particular, the Pearson correlation coefficient of *p*_epitope_ with H3N2 vaccine effectiveness in humans is *R*^2^ = 0.75. Interestingly, this correlation is nearly the same as that previously reported for the 1971–2004 subset of years (Gupta *et al*., 2006), despite the addition of 50% more data. Also of significance to note is that these correlations with *p*_epitope_ are significantly larger than those of ferret-derived distances with vaccine effectiveness in humans over the period 1968–2015, which are *R*^2^ = 0*.*39 or 0.37 for the two most common measures (Table I and Gupta *et al*., 2006).

### Consistency of epitopic sites

Analysis of HA1 sites shows that of the sites under diversifying selection (Pan and Deem, 2011), there are only 10 that by this measure should be added to the 130 known epitope sites (Gupta *et al*., 2006). Alternatively, of the sites under diversifying selection, 81% are within the known epitope regions (Pan and Deem, 2011). The 130 epitope sites that we have used nearly cover the surface of the head region of the HA1 protein, and this is why they are nearly complete. Another recent study (Meyer and Wilke, 2015) identified epitopes somewhat different from those that we use and further suggested that proximity to receptor binding site is a significant determinant of H3 evolution. Distance from the sialic acid receptor-binding site is significant because the sialic acid receptor-binding site is in epitope B, which is adjacent to epitope A, and epitopes A and B are the most commonly dominant epitopes over the years (Table I and Table 1 of Gupta *et al*., 2006). We note, however, that upon computing the correlation of *p*_epitope_ using the four epitope sites defined in Meyer and Wilke (2015) with the human vaccine effectiveness data considered here, one finds *R*^2^ = 0.53. This result is to be compared with the *R*^2^ = 0.75 illustrated in Fig. 2.

### The influenza A/H3N2 2014/2015 season

The 2014/2015 influenza vaccine contains an A/Texas/50/2012(H3N2)-like virus to protect against A/H3N2 viruses (World Health Organization, 2014a). Novel viral strains detected in the human population this year include A/Washington/18/2013, A/California/02/2014, A/Nebraska/4/2014 and A/Switzerland/9715293/2013 (World Health Organization, 2014b). It should be noted that A/California/02/2014 and A/Switzerland/9715293/2013 are completely identical in the HA1 sequence that contains the HA epitopes. (There is one substitution each in epitopes A, B, and D for the A/Switzerland/9715293/2013 E4/E2 strains.) Table II shows the *p*_epitope_ values between the vaccine strain and these newly emerged strains. The values indicate, along with Fig. 2, that the vaccine is unlikely to provide much protection against these strains because *p*_epitope_ > 0*.*19.

**Table II.** The *p*_epitope_ distances between the vaccine strain A/Texas/50/2012(egg) and selected novel strains

Strain name	Collection date	*p_i_* for each epitope *i*						Predicted effectiveness
		A	B	C	D	E	*p*_epitope_	
A/Texas/50/2012(cell)	2012-04-15	0	0.0476	0	0.0244	0	0.0476	35%
A/Washington/18/2013	2013-11-29	0.1053	0.1905	0	0.0244	0	0.1905	0%
A/California/02/2014	2014-01-16	0.1579	0.1905	0	0.0244	0	0.1905	0%
A/Nebraska/04/2014	2014-03-11	0.1053	0.2381	0.0370	0.0244	0.0455	0.2381	0%

The *p*_epitope_ distances between the vaccine strain A/Texas/50/2012(egg) and reported novel strains (World Health Organization, 2014b) in 2013 and 2014. The *p_i_* values for each epitope (*i*= A–E), the number of substitutions in epitope *i* divided by the number of amino acids in epitope *i*, are also shown. The value of *p*_epitope_ is the largest of the *p_i_* values, and the corresponding epitope *i* is dominant. Zero values indicate no substitutions in that epitope.

### Dynamics of influenza evolution

**Fig. 3:**
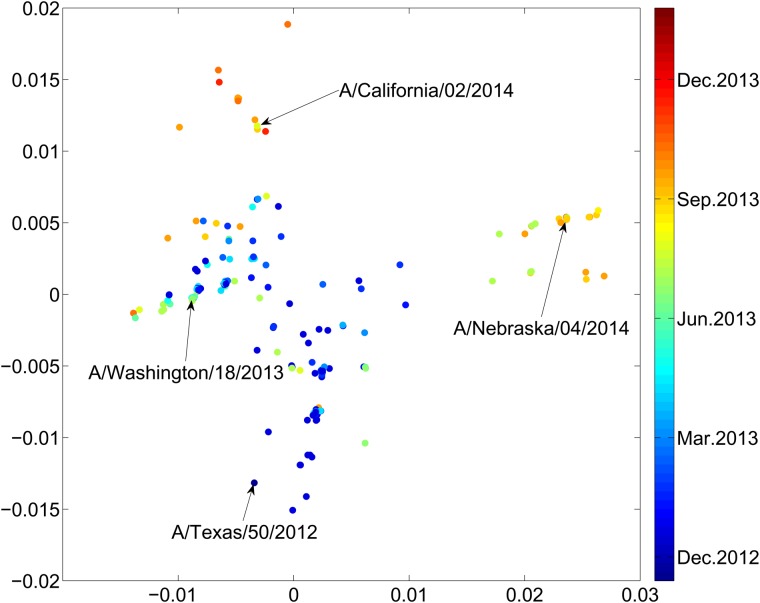
Dimensional reduction of all H3N2 influenza sequences collected from humans in 2013 and 2014 and deposited in GenBank. Distances are normalized by the length of the HA1 sequence, 327 aa. Dimensional reduction identifies the principal observed substitutions, i.e. those correlated with fitness of the virus, which we expect to be in the epitope regions. A value of *p*_epitope_ = 0.19 corresponds to a distance of 0.012 here. Sequences from Table 2 are labeled. While the A/Texas/50/2012 sequence was collected in 2012, substantially similar strains were collected in 2013 and downloaded from GenBank.

The strains detected in 2013 and 2014 cluster in sequence space. While the strains are sparse in the full, high-dimensional sequence space, this clustering is detected by multidimensional scaling to the two most informative dimensions, as shown in Fig. 3. The novel strain A/Washington/18/2013 emerged in 2013, followed by A/California/02/2014 and A/Nebraska/4/2014 in 2014, as shown in Fig. 3. The latter two are sufficiently distinct from previous vaccine strains that expected vaccine effectiveness is limited.

**Fig. 4:**
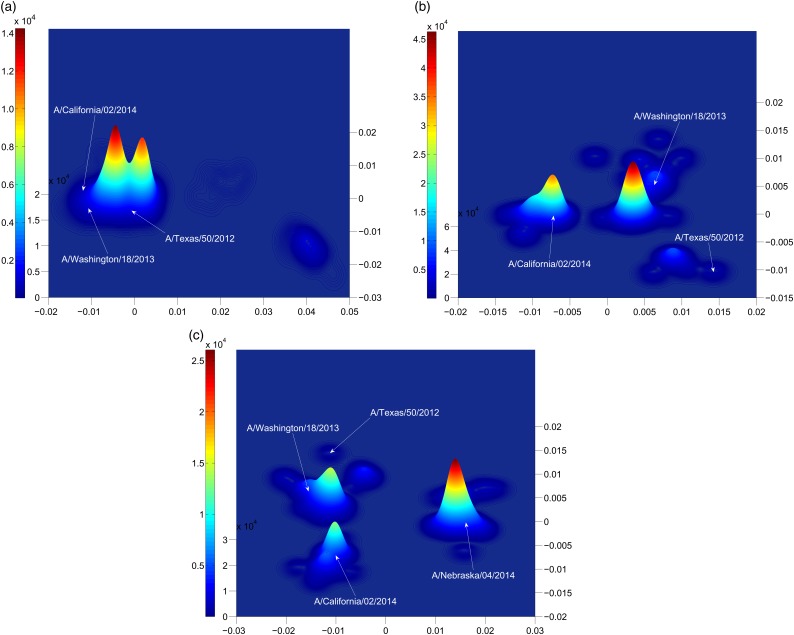
Gaussian density estimation of sequences in reduced two dimensions for (**a**) all 2013 H3N2 influenza sequences in humans, (**b**) those 2014 H3N2 influenza sequences in humans near the A/Texas/50/2012 sequence, and (**c**) all 2014 H3N2 influenza sequences in humans. The consensus strain of the cluster to which A/Nebraska/4/2014 belongs is A/New Mexico/11/2014.

Figure 4 is an estimate of the density distribution of the influenza H3N2 HA1 sequences in years 2013 and 2014 in the low-dimensional space provided by the multidimensional scaling. Dimensional reduction was applied to the subset of sequences in each subfigure (Fig. 4a, b or c). Then, Gaussian kernel density estimation was applied to estimate the distribution of sequences in the reduced two dimensions. Each sequence is represented by a Gaussian function with a standard deviation of one-half substitution in the dominant epitope.

By the criteria above, A/California/02/2014(H3N2) represented the dominant strain circulating in the human population in 2014/2015. The time evolution in Fig. 3, or a comparison of Fig. 4a with Fig. 4b, shows that the A/California/02/2014 cluster emerged in 2014. Table II shows that the distance of this new cluster from the A/Texas/50/2012(egg) strain is *p*_epitope_ > 0*.*19, and so from Fig. 2 the expected effectiveness of A/Texas/50/2012(egg) against these novel A/California/02/2014-like strains is zero. Conversely, an effective vaccine for this cluster in the 2014/2015 flu season could be A/California/02/2014, or the A/Switzerland/9715293/2013 that is identical in the HA1 region.

### Early detection of new dominant strains

Surprisingly, when we enlarge the region of sequence space considered, going from Fig. 4b to Fig. 4c or 3, we find another large and growing peak at a distance *p*_epitope_ = 0.24 from the A/Texas/50/2012 sequence. This new cluster contains the A/Nebraska/4/2014 sequence. The A/Nebraska/4/2014 sequence is *p*_epitope_ = 0.16 from the A/California/02/2014 sequence. The A/Nebraska/4/2014 sequence appears to be dominating the A/California/02/2014 sequence in the 2015/2016 season. The consensus strain of this cluster to which A/Nebraska/4/2014 belongs is A/New Mexico/11/2014. The consensus strain minimizes the distance from all strains in the cluster, thus maximizing expected vaccine effectiveness. Thus, A/New Mexico/11/2014 might be a more effective choice of vaccine for the majority of the population in comparison to A/Switzerland/9715293/2013 or A/California/02/2014.

### Phylogenetic analysis

A systematic phylogenetic analysis of recent A/H3N2 virus HA nucleotide sequences has been carried out (McAnerney *et al*., 2015; Stucker *et al*., 2015). Briefly, phylogenetic trees were reconstructed from three reference sequence datasets using the maximum likelihood method (Stucker *et al*., 2015), with bootstrap analyses of 500 replicates. Dominant branches of the tree were identified with distinct clade labels. Analysis of the HA protein sequences showed that there were relatively few residue changes across all HA clades. The 2014 vaccine strain A/Texas/50/2012 falls into clade 3C.1, while the new emerging A/California/02/2014 strain falls into subclade 3C.3a. The A/Nebraska/4/2014 and the consensus A/New Mexico/11/2014 strains fall into subclade 3C.2a. The phylogenetic analysis indicates a closer relationship of A/Nebraska/4/2014 or A/New Mexico/11/2014 to A/California/02/2014 than to A/Texas/50/2012.

Note that phylogenetic methods make a number of assumptions. For example, substitution rates at different sites are assumed to be the same and constant in time. Due to selection, however, substitution rates are dramatically higher, perhaps 100×, in dominant epitope regions than in non-dominant epitope or stalk regions. Multigene phylogenetic methods are inconsistent in the presence of reassortment, and single-gene phylogenetic methods are inconsistent in the presence of recombination, with the former being perhaps more significant than the latter in the case of influenza. Multidimensional scaling, on the other hand, does not make either of these assumptions. MDS also naturally filters out neutral substitutions that are random as the dominant dimensions are identified. Thus, MDS provides a complementary approach to the traditional phylogenetic analysis.

### Ferret HI analysis

Since an analysis showing that the correlations between the two standard methods of analyzing ferret hemagglutinin inhibition antisera assays with vaccine effectiveness in humans in the years 1968–2004 were *R*^2^ = 0.47 and 0.57 (Gupta *et al*., 2006) first appeared, a number of studies have reported results supporting these low correlations. For example, Table 3 of Ansaldi *et al*. (2010) shows that correlation of various immunogenicity parameters is higher with genetic distance than with HI measures of antigenic distance. The study by Xie *et al*. (2015) further illustrated the limitations of relying on ferret HI data alone. We have updated our calculation of the correlations between the two standard methods of analyzing ferret hemagglutinin inhibition antisera assays with vaccine effectiveness in humans to the years 1968–2015, see Gupta *et al*. (2006) and the last two columns of Table I. The correlations with *d*_1_ and *d*_2_ are now *R*^2^ = 0.39 and 0.37, respectively, showing that ferret HI studies have become even less correlated with human vaccine effectiveness in recent years.

## Conclusion

We have shown how vaccine effectiveness can be predicted using *p*_epitope_ values. This method requires only sequence data, unlike traditional methods that require animal model data, such as ferret HI assay experiments or post-hoc observations in humans. Interestingly, the correlation of *p*_epitope_ with H3N2 vaccine effectiveness in humans is *R*^2^ = 0.75, nearly the same as that previously reported for the 1971–2004 subset of years (Gupta *et al*., 2006), despite the addition of 50% more data. The correlation of H3N2 vaccine effectiveness in humans with *p*_epitope_ is significantly larger than with ferret-derived distances, which are *R*^2^ = 0.37 or 0.37 for the two most common measures (Gupta *et al*., 2006). As an application, we estimated the effectiveness of the H3N2 vaccine strain of A/Texas/50/2012 against the observed A/California/02/2014 strains.

Clustering of the 2013 and 2014 sequence data confirms the significance of the *p*_epitope_ measure. We showed from data through 2014 that there is a transition underway from the A/California/02/2014 cluster to a A/New Mexico/11/2014 cluster. The consensus sequence of A/New Mexico/11/2014 from this cluster could have been considered in late Winter 2015 for inclusion among the H3N2 candidate vaccine strains for the 2015/2016 flu season.
